# Academy of Stomatology

**Published:** 1901-10

**Authors:** 

**Affiliations:** Academy of Stomatology


### ACADEMY OF STOMATOLOGY.

A regular monthly meeting of the Academy of Stomatology was held at the rooms of the Academy, 1731 Chestnut Street, Philadelphia, on the evening of Tuesday, June 25, 1901, the President, Dr. S. B. Luckie, in the chair.

After the transaction of routine business Professor E. T. Darby addressed the Academy upon the subject of "Instrument Handles."

### INSTRUMENT HANDLES.

BY DR. E. T. DARBY, PHILADELPHIA.

It was suggested to me a short time ago that as manufacturers are at a loss to know whether dentists prefer long handles or socket handles, they often are overstocked with one kind or the other; also that as students often do not know what to select in this line, it would be well to call up a discussion upon this point. The evolution of dental instrument handles is interesting, as is that of many other things in the practice of dentistry. In olden times the outfit of the dentist consisted of comparatively few instruments, and these few were, I think, what we would consider to-day clumsy of construction and badly adapted to our present mode of practice. The dental outfit could then be put into a box perhaps eighteen inches long, a foot wide, and six or seven inches high. You have doubtless all seen the equipment of the dentist of forty or fifty years ago. The instruments were fitted into a series of plush- or velvet-lined trays. They were not numerous, nor greatly varied in shape. The handles, which were made of ivory or mother-of-pearl, often ornamented with gold ferrules and carving, were apparently intended as much to impress the patient as to serve for operations upon the teeth. I do not know how I worked with instruments of that description with any degree of comfort, and am sure few would care to return to their use. It is true, we packed gold by hand-pressure, grasping the instruments in the palm of the hand. In working on the inferior jaw great pressure was often used in packing gold. I doubt if the average dentist to-day would use an instrument with a handle as heavy and large as those we used, except perhaps for burnishing or other great force. As in evolution, the handle was reduced, the size of the point was reduced, and this was marked when mallet force took the place of handle-pressure for packing gold. I remember very distinctly when, in 1862, Dr. Atkinson, of New York, constructed the first set of mallet-pluggers that were known to the profession. It consisted of thirty-four or thirty-six instruments with long, slim handles, and were intended for mallet use. From that day I almost entirely discarded the heavy handle instrument and made use of the mallet style.

Owing to my preceptor's influence, I early formed the habit of making my own instruments, and it became a pleasure to me, and during the earlier years of my practice I made a great many for my own use. Most of these instruments which I exhibit here I made many years ago.

With regard to the relative advantage of the long or solid handle and the socket handle, there is, of course, a difference of opinion. The socket handle is intended to effect an economy in the points which screw into it. This way of using an instrument is certainly the more economical. The point of the instrument wears down, and can only be renewed a certain number of times, because the instrument becomes too short and the quality of steel is much impaired by frequent heating and loss of its carbon; therefore the instruments become really almost worthless.

I was led some years ago to adopt a uniform style of socket handle in my own practice, having learned the fact that I became accustomed to a certain-sized handle for an excavator and another for a plugger, and was embarrassed by a change from one size to another during the same class of work; as, for instance, one put into a large handle a small excavator point. If, on the other hand, one put a large point, or large burnisher, or an instrument with a right angle, into a small-handled instrument, he would find he was working to a disadvantage by being unable to grip the handle firmly enough. For packing gold, handles of uniform size, that were smooth and yet not slippery like nickel-plate, satisfied me and felt more comfortable in my hand than one that was roughened. So I made for myself a large number of snake-wood handles and some of ivory, and tried to make them of uniform and appropriate size, which one can determine by the feeling imparted to the hand.

At that time we had no cone-socket handles, and I conceived the idea of drilling these instruments to a certain depth, which I accomplished by using a guard on my drill, which permitted a penetration of only three-eighths or half an inch. Each one is drilled to the same depth and reamed with the same reamer, so that every one of the points will fit securely every one of the set of instruments, by reason of the exact taper of both shank and socket. With the cone-socket points, unless set firmly in the handle, they are liable to turn or unscrew, just in proportion as the point of the instrument is inclined away from a direct line with the shank of the instrument. They can be securely fixed in the handles by means of a pair of pliers, a specially recessed pair preferred.

In proportion as the point is small, the handle should be light and small. Let me illustrate that. If I take an instrument used in the canals of teeth and put it into a large handle, I would work to great disadvantage, constantly feeling something in my hand unwieldly and uncomfortable. If, on the other hand, I mount it in a light, delicate handle, so that I do not feel the weight, my touch is lighter and steadier. To make these light handles hold the points securely and yet be of very little trouble in making, I drilled them to a certain depth and tapped them. The points of piano wire were soft soldered into a drill hole in the end of a piece of German silver, threaded on either end to fit the tap-hole in the handle. The soft soldering does not draw the temper of the wire. This makes an instrument delicate to the touch and light in weight, and where the point is broken or it needs replacing it is very easily replaced. The way of setting the broaches into shellac was a clumsy way and not satisfactory. These little piano-wire points are made by grinding the wire down on a two-inch corundum-wheel which has a slight groove worn in the centre of its grinding face. Water is allowed to constantly drip upon the stone, so that the point can never be heated, though the lathe runs at a great speed. To make a hook on it, I bend the end in a pair of pliers, and if the point is too long I rub it on an Arkansas stone and shape it as I please; and so on with a variety of forms of piano-wire instruments.

The chisel is an instrument that is used by most practitioners. If it is not, it ought to be. The greatest trouble with chisel-handles is that you do not have an opportunity of grasping that chisel with a firm hold. To that end I have made my chisel-handles, many of them, short and with a bulbous end, somewhat like an engraver's tool, so that it will not hurt the palm of my hand.

Going back to the relative advantages of handles, I think it is a matter that every dentist must decide for himself. I do not think I could say that a long-handled instrument would be more satisfactory to you than a short or socket handle; at the same time there are disadvantages in the socket handle. The first I have already noted,—that the point is liable to loosen in it; that can be overcome in the way I have suggested. On the other hand, there is perhaps a saving in the point. Though, if you put the aggregate cost of the point and socket together, you will find that your cone-socket, and the point that goes with it, costs more than the long-handled instrument. The instrument-makers prefer selling the instruments with long handles. Most of the instruments that are made at the present time are so infinitely better than those of twenty or thirty years ago, that the student or practitioner can go into a dental depot and can find almost anything that he wants well made and well tempered. We could formerly get burnishers and large chisels very well tempered, but it was a most difficult thing to get small instruments and excavators well tempered and well shaped.

It seems to me that it is of importance that students, especially those who have plenty of time, should know something about the manufacture and a good deal more about the tempering of instruments. Very often a man wants an instrument of a certain bend or curve or angle. If he knows how to shape and temper steel, he can go into his laboratory and in a few minutes make it. That advantage is tenfold greater when a man is located far away from a supply house.

As I said at the outset, this subject was suggested to me, and I am therefore simply in the position of starting a discussion of this subject.

DISCUSSION.

*Dr. Guilford.*—The point made in regard to the delicacy of touch was well taken. The handles of my cutting instruments are made of steel, and are such as we buy, but with the pluggers I almost invariably use the wooden handles. I buy the wooden handles and make them to suit me, or buy them ready-made. I do that for the reason that with the wooden handle in my hand I can feel where the instrument is going by the sense of touch much better than I could in any other way. There is one place where we particularly need to take advantage of that sense of touch, and that is in the removal of the pulp. I do not see how any one can undertake to explore the canal of a tooth or root, and remove the pulp and feel his way when he has a steel instrument in his hand. It does not make any difference how lightly it is made, it is still heavy. The wood conveys the vibrations to your hand. There is a handle of that kind made for that purpose which has a little chuck on the end of it; the rest of it is made of wood and I have great satisfaction in using it. A metal handle for pluggers and light work is not satisfactory. Even ebony or vulcanite is too heavy; some lighter wood, made of a size to fit the hand, is better. The sense of touch should be cultivated in the dentist exactly as it is in the physician who depends upon it. I agree entirely with all that Dr. Darby has said on this subject.

*Dr. William H. Trueman.*—The handle of an instrument is a matter of personal equation. On reading the announcement on the card, I was reminded of a friend. He had a very large hand, and said that the small instruments were uncomfortable. He wanted something to fill up his hand, and he had an entire set made with the handles from a half to three-quarters of an inch in diameter.

I agree with both points that Dr. Darby has laid down. We should have the point in correspondence with the handle. If the point of the instrument be small and delicate, we want the handle to correspond so as to feel comfortable and not cramped. Since microbes have been around I have discarded nearly all the instrument-handles except those of steel. I can put them into hot water with no danger of their coming apart, and if the water is very hot there is no danger of spoiling the handle. With the exception of a very few, I desire to have a large handle, so as to get a firm grasp upon it and to be able to use considerable pressure in rotating.

I have them classified, and have a different handle for each class. The hoe excavators have a groove cut into one end of the handle. And when I see them lying in a case I know whether a certain one is a hatchet or a hoe excavator. The same way with my burnishers and my pluggers: each one has a different handle, so that I recognize it at a glance. Other instruments are silver-plated, to distinguish them from the nickel-plated ones. When I see an instrument anywhere, I know what instrument it is without having to look at the point. That was impressed on me by once having used an excavator for a plugger by mistake. I discarded roughened handles, as they made my fingers sore, and used the simple, smooth handle, and had them all nickel-plated, and I find I am not troubled with slipping.

The socket points I have discarded almost entirely. I use an instrument that has more solidity about it. I was troubled with the turning around that Dr. Darby spoke of, and adopted the plan of using shellac to fasten them in; a very little put on makes it perfectly firm and it can be readily removed; but the microbes have interfered with that again, for the hot water will, in time, loosen it. It is very interesting to observe the changes which the instrument-handles have undergone.

Some of the older instruments were beautifully made and nicely finished with a great many curves and angles, and very beautiful to look at, but were very uncomfortable to use. A great many of them were ornamented with precious stones. I remember a set gotten up by Dr. Plato. The instrument-case was a small one, not more than one foot square, and the instruments alone cost fifteen hundred dollars. I saw them not very long ago, having been called upon to appraise their value. There was not an instrument in the case that any one of us would accept if we were compelled to use it. They had cameo handles, and were set with various kinds of jewels, but the very fact of the handles having such precious stones would interfere with their use. The simplest instrument that we can get, one that will not be injured by falling upon the floor, is the best.

*Dr. Gardiner.*—I do not know that I can add very much to what has already been said. I agree with Dr. Trueman as to the personal equation, and think that the instruments which one has been taught to use in the beginning are very likely to be the ones which will be approved of. It is very difficult for any one individual to select a handle which will suit every one equally well, because of individual peculiarities. For myself, I use nearly all styles of handles for different kinds of instruments. I have a great variety of them, and I like each one in its place. Dr. Darby has shown a great deal of ingenuity and skill in the manufacture and in the design of these handles, which seem admirably adapted to the purpose.

*Dr. James Truman.*—The evolution of the dental instrument has always been interesting to me, perhaps for the reason that I have gone through much of this experience. I ofttimes looked at Dr. Darby's instruments and wished that I had his skill when I was in practice, although I made a large number of my own instruments. In fact, in the earlier days of dentistry in this country it was necessary that every dentist should have some knowledge of the formation of instruments. It was almost impossible to get these properly formed. About 1850 all the instruments that were procurable were made by the surgical-instrument-makers, and their tempering of the excavators, especially drills, was very bad. We had to re-temper, or make our own instruments, especially excavators, in order to get any good results. They were tempered so hard as to become dangerous in the hands of the dentist.

I cannot quite agree with Dr. Darby that the dentists of that period—that is, the better dentists—had these large-handled instruments. I know it was common to a certain class of operators to have large cases with splendidly jewelled instruments, and all that sort of thing, but I was under the instruction of Dr. Elisha Townsend, and he never had any such instruments, but all small-handled instruments. I myself never used at that earlier period any such instruments as have been described by my friend Dr. Wm. Trueman,—large-handled instruments for packing soft gold. Perhaps that was because they were not comfortable in my hand. I could get sufficient force from my fingers without the force from the whole wrist. That seemed to me a barbarous way of putting in fillings, even by the soft-foil method.

The evolution of instruments, from the period when smooth, round points were used in packing non-cohesive gold, to that of serrated points, is one of interest, for it indicates the progress made towards the introduction of cohesive gold which came in later. The increase in the serrations was gradual, but they became so marked in character as naturally to produce a reaction, and we are now back again to almost smooth points, which I regard as a mistake.

I agree fully with Dr. Darby in regard to the handles. I think he has made an excellent form of handle, but do not like the cone-socket, that is, the screw passing into the instrument. I agree with him that they are liable to become loose, but I cannot agree with Dr. Wm. Trueman in putting in shellac to hold them. That is not my idea of an instrument. I want a firm instrument. I should prefer all instruments solid from one end to the other.

*Dr. Hickman.*—The paper has been very interesting, and I am always glad to listen to remarks upon the tempering of instruments. Dr. Guilford spoke of wooden handles for broaches for the removal of pulp, which rather surprises me. Somebody taught me to use a broach without a handle, and even to cut off part of the shank. That might have been Dr. Darby. He did not teach us to use handles then. I cannot find anything better than that. When you get just as near to the pulp as you can without a shank, you can judge by the tactile sense.

*Dr. Allen.*—In a collection of instruments that had not been used for sixty years, I came across a few oddities which I purchased as objects of interest. I have here a few samples of the large handles referred to by Dr. Trueman. You can see the immensity of the instruments, which have never been used, and, I believe, have been lying idle for sixty years. With the same outfit is a unique mouth-mirror scarcely a thirty-second of an inch wide, capable of doing work on each side of the mouth. I also have a couple of handles used for the last forty years daily for the introduction of amalgam fillings. These were the instruments of Dr. Bonwill, solid, strong, and as durable as the fillings introduced by him. They have been used so much that the serrations are almost worn off. Notice the uniqueness of the points. They are exceptionally strong and heavy. They were used by Dr. Bonwill on almost every filling that he put in.

*Dr. Grubb.*—As one of the younger members of the profession, I appreciate these opportunities to gain some knowledge from the experience of such men as Dr. Darby. I remember when at college I received information concerning the tempering of instruments, and it has been of as much use to me as anything that I ever acquired there in the way of instrument-use. The idea of the instrument-handles and points being in proportion to the surface they are brought against is something I have always followed.

*Dr. Register.*—There is no doubt in the world that different operations require different handles, for the very reason that many of the operations that we are performing require different amounts of force. In other operations we want to have a delicate touch, and you cannot get that with a large handle. I have been very much impressed recently with the necessity in certain operations of using small handles in connection with small instruments. Take, for example, instruments for the treatment of pyorrhœa. I do not see how any large instrument will help us much in the treatment of that disease. We want small instruments and we want small handles. The instruments that Dr. Darby has passed around this evening simply fill me with admiration. Certainly there is nothing in the office of a dentist who wants to do artistic work that is of as much help as a handle well adapted to the operation to be performed.

Without that I think many operations fail that otherwise would succeed.

*Dr. Woodward.*—I prefer the small instruments of steel to the larger instruments of wood, ebony, or ivory. Here are two double-ended instruments; the handles were made by Dr. Hollingsworth, of the S. S. White Dental Manufacturing Company. The four points I designed myself. I have ordinarily a prejudice against an instrument with two points. I want an instrument with only one point. But these burnishers work so beautifully, and can be reversed so quickly, that I have become attached to them.

*Dr. Gaylord.*—I am an admirer of the Darby-Perry spoon excavators, and like a double-ended handle. I put the right-hand instrument in one end and the left-hand instrument in the other, and find it very convenient to excavate from one direction, and, by turning the instrument in my hand, work in the other direction. It is a saver of time and handles; you know where the mating instrument is. The idea is applicable to many of the plastic filling-instruments. I have selected and purchased different knobs for each class of instruments, which aids in finding them. I am using the metal handles entirely, except in the pluggers, simply because I can boil and sterilize them. The gold instruments being used with the rubber dam on the mouth, I do not consider that they should be boiled.

*Dr. Darby.*—I have very little to say in closing. I neglected to speak of one point, and that was that in order to distinguish between plugging and excavating instruments, I have had the former gold plated. That only applies to the smaller plugger-points; the larger ones I use in the snake-wood handles, etc. My objection to a double-ended instrument is that one end may wound the palm of the hand. I got several wounds from a double-ended instrument in this way, and from an infected excavator that would be a serious matter.

Adjourned.

OTTO E. INGLIS,
*Editor Academy of Stomatology.*

